# Older cancer survivors living with financial hardship in China: The Influence of Confucian Family Values

**DOI:** 10.1093/geroni/igab046.3030

**Published:** 2021-12-17

**Authors:** Mingzhu Su, Li Liu, Hanlin Yue, Jiajun Zhang, Nengliang Yao

**Affiliations:** Shandong University, Jinan, Shandong, China (People's Republic)

## Abstract

Background: Financial hardship has not been well studied among older cancer survivors, despite its debilitating effects on their health and well-being. Aim: To describe the lived experience of older Chinese cancer survivors and explore the financial impacts following a cancer diagnosis. Design: A qualitative study conducted using semi-structured interviews with patients and family caregivers. Methods: We individually interviewed twenty-one cancer survivors (aged □ 60) with financial hardship and twenty family caregivers in Shandong province between August 2020 and January 2021. A content analysis was performed by multiple coders. Findings: Confucianism culture and the Chinese health system considerably impact the construct of financial hardship and its components. Four main categories were revealed:(1) healthcare providers were reluctant to discuss the diagnosis and costs of care with cancer patients; (2) financial transfer from adult children to older parents became prevalent after a cancer diagnosis;(3) cancer-related financial worries and stress spilled out into children's family; (4) coping and adjustment strategies were taken by the extended family. Conclusion: Both older cancer survivors and their adult children experienced financial distress mediating through filial piety in China. Instruments are needed to screen for cancer-related financial hardship adapted to the healthcare system and Confucian family values. Key words: Cancer survivors; older; financial hardship; qualitative; China

